# Public attitudes in Japan toward the creation and use of gametes derived from human-induced pluripotent stem cells

**DOI:** 10.2144/fsoa-2021-0066

**Published:** 2021-10-22

**Authors:** Tsutomu Sawai, Taichi Hatta, Kyoko Akatsuka, Misao Fujita

**Affiliations:** 1 Institute for the Advanced Study of Human Biology (WPI-ASHBi), KUIAS Kyoto University, Japan, Yoshida-Konoe-cho, Sakyo-ku, Kyoto, 606-8501, Japan; 2 Shizuoka Graduate University of Public Health, Japan, 4-27-2 Kita Ando, Aoi-ku, Shizuoka, 420-0881, Japan; 3 Uehiro Research Division for iPS Cell Ethics, Center for iPS Cell Research & Application, Kyoto University, Japan, 53 Kawahara-cho, Shogoin, Sakyo-ku, Kyoto, 606-8507, Japan

**Keywords:** assisted reproductive technologies, concerns, degree of acceptance, ethics, expectations, human-induced pluripotent stem cells, *in vitro* derived-gametes, in vitro gametogenesis, public survey

## Abstract

**Aim:**

To ascertain to what extent the Japanese general public accept the creation and use of *in vitro* derived (IVD)-gametes derived from human-induced pluripotent stem cells.

**Materials & methods:**

We conducted an online survey and obtained answers from 3096 respondents.

**Results:**

78.6% of the respondents answered that they would accept the creation and use of IVD-gametes for research purposes, 51.7% answered that they would accept the creation and use of embryos with IVD-gametes for research purposes, and 25.9% answered that they would accept childbirth using embryos with IVD-gametes.

**Discussion:**

The results that approximately half of the respondents answered that they would accept the creation of embryos with IVD-gametes, which has not been allowed in the current Japanese research guidelines, is astonishing.

## Lay abstract

This paper reports the results of an online public survey about the creation and use of *in vitro* derived (IVD)-gametes, conducted in Japan with 3096 respondents selected from the monitor members of a survey company. While IVD-gametes generate interest for research purposes as well as reproductive use, ethical issues have been discussed in relation to such usage. It is of value to understand the extent to which the creation and use of IVD-gametes are considered acceptable by the general public and the expectations and concerns they may have, which will inform future discussions on regulations of this technology.

In recent years, research progress has been made in creating *in vitro* derived (IVD)-gametes from human pluripotent stem cells, including human-induced pluripotent stem cells (hiPSCs) and human embryonic stem cells (hESCs). In mice, Hayashi *et al.* have reported the generation of offspring from sperm and ova derived from mouse pluripotent stem cells (PSCs) [1,2]. Furthermore, although the creation of IVD-gametes has not been achieved in humans at present, Irie *et al.* created primordial germ cell-like cells from human pluripotent stem cells in 2015 [3], and Sasaki *et al.* successfully created primordial germ cell-like cells from hiPSCs through more efficient procedures in 2015 [4]. Furthermore, in September 2018, Yamashiro *et al.* reported the successful generation of oogonia, which finally differentiate into ova, from primordial germ cell-like cells derived from hiPSCs [5].

The creation of IVD-gametes and their use for research would help acquire biological insights into human development, to elucidate the etiologies of infertility and genetic diseases, and to establish therapeutic modalities for these conditions [6]. The creation of IVD-gametes would also enable the procurement of unfertilized/fertilized ova, which has so far been difficult, for research purposes [7]. Furthermore, the use of IVD-gametes for reproductive purposes would pave the way for those who cannot have children with the current assisted reproductive technology (ART), including couples suffering from infertility, persons who are congenitally infertile for conditions such as congenital disturbance of spermatogenesis, and individuals who lose their fertility owing to cancer treatment and other noncongenital reasons, to have genetically related children [8]. Thus, *in vitro* gametogenesis (IVG) technology is expected to bring many benefits to both research and reproduction.

However, ethical issues have also been raised from the beginning with regard to the creation and use of IVD-gametes [8,9] For instance, it is theoretically possible to produce as many embryos as needed from embryos with IVD-gametes; however, unlike in the case of using the embryos that were created for infertility treatment but were finally not used (‘surplus embryos’) and then donated for research, the theoretical possibility of creating embryos with IVD-gametes poses the following question: “Is it acceptable to produce and use embryos solely for research purposes (‘research embryos’) in the first place?” [7]. In addition, the use of IVD-gametes for reproductive purposes poses the problem of safety risk for the children to be born and the future generations [10], and the question of to whom and for what purposes the use of IVG technology should be permitted [7,11–14].

For IVG technology, which is thus controversial, in May 2010, the Japanese government established research guidelines that allow the creation of IVD-gametes [15]. Later, in 2015, the Expert Panel on Bioethics of the Cabinet Office resumed the discussion on whether to allow the creation of embryos with IVD-gametes; however, deregulation was postponed on the grounds that it was inappropriate to judge the necessity of creating embryos with IVD-gametes at the stage where the creation of IVD-gametes had not yet been achieved [15–17]. In contrast, the Japanese government has allowed the limited use of research embryos created without using IVD-gametes, only in research expected to contribute to the advancement of ART [18].

In other countries, no special regulations have been established for research using IVD-gametes and embryos with IVD-gametes [19], and such research is likely to be subject to the general regulations for research using human embryos. For instance, the US NIH guidelines prohibit federal funding of research that might result in the destruction or risk of damage to human embryos with the exception of utilizing surplus embryos to create hESCs [20], but the statutes enacted in California allow the creation of embryos for research purposes. Many European countries, except the UK, Denmark, Sweden and Belgium, ban the creation of embryos for research purposes.

The creation and use of IVD-gametes can affect not only individuals but also the whole society and even future generations. Once IVG technology is established, the issue of discussion will be ‘how should IVD-gametes be used ethically in research and clinical settings’ rather than ‘whether the creation and use of IVD-gametes should be permitted’. Therefore, it is necessary to discuss with potential stakeholders who are likely to be affected by the creation and use of IVD-gametes to what extent they should be permitted before the creation of IVD-gametes is achieved [21,22]. The aim of this study was to elucidate using a questionnaire survey what expectations and concerns the Japanese general public, who constitute the stakeholders, have with respect to the creation and use of IVD-gametes, as well as the extent to which they accept a series of processes involving research using IVD-gametes to their clinical use.

## Materials & methods

### Selection of respondents

The study was conducted in an online-only format because we considered this to be a suitable method to survey the opinions of a group of people representative of the Japanese population (in terms of age and gender) simultaneously. In this survey, we prepared a questionnaire and then outsourced the data collection to a survey company (a confidentiality agreement is in place with the survey company prohibiting the disclosure of the company name and details). To recruit the participants of the online survey, the survey company sent an e-mail on 17 May 2017, to all the monitor members who were between 20 and 80 years of age. The survey company asks monitor members to update their personal information once a year, such as addresses, annual incomes, and other sociodemographic attributes. Our study specified that the recruiting conditions included only those individuals living in Japan; thus, we can reasonably assume that all the respondents were residents of Japan at the time of the survey.

The e-mail contained the URL of the webpage for informed consent of the survey. The webpage specified the aim of the survey, the estimated time required for answering (10–15 min in total), remuneration for participation (i.e., redeemable points issued by the company; for confidentiality reasons, the incentives cannot be explained in greater detail), that personal information would be handled according to the company’s policy, that the answers would be anonymized, and the information regarding the executive agency of this survey. The ‘Next’ and ‘Cancel’ buttons were placed at the bottom of the webpage. Clicking the ‘Next’ buttons was considered as giving consent: volunteer opt-in panels [23]. On 18 May 2017, the number of participants reached the necessary sample size. The company closed the URL and terminated the recruitment of participants and the collection of answers on the same day. Therefore, this procedure does not yield valid response rates.

While the data collection procedure was based on the non-probability sampling strategy, the sample sizes were designed based on the probability sampling [23]. In general, the minimum sample size for a public opinion poll is estimated to be 384 (confidence level: 95%; margin of error: 5%; and response rate: 0.5%). To grasp the attitudes of the Japanese general public, we classified the Japanese general public aged 20–79 years into six age groups set at a constant interval (20–29, 30–39, 40–49, 50–59, 60–69 and 70–79 years old). We assigned 384 people to the 20–29 age group as the least populated group based on the latest available demographic statistics [24], and estimated the number of respondents in each group 384, 481, 543, 463, 546 and 415, respectively (2832 in total). Minors younger than 20 years and elderly people aged 80 years or older were excluded from the survey. Complete responses were obtained from 3096 respondents, which exceeded the required sample size due to its web platform specifications. The distribution of age groups and sex was consistent with demographic data.

### Content & questionnaire preparation procedures

**Figure 1.**
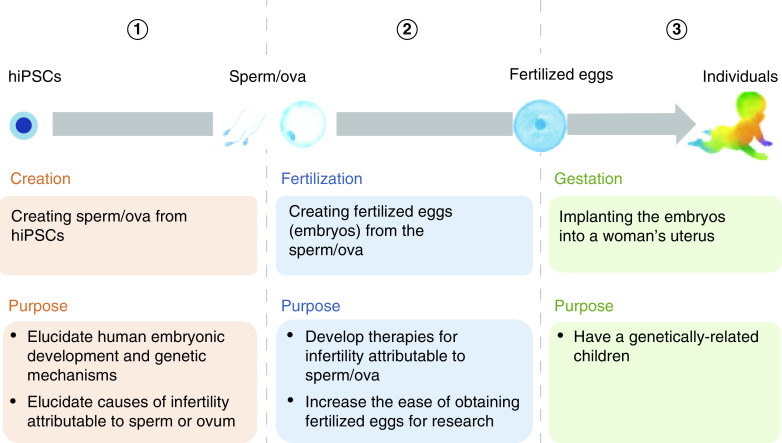
Process of creating and using human-induced pluripotent stem cells-derived sperm/ova. hiPSCs: Human-induced pluripotent stem cells.

This questionnaire is part of a survey project investigating the opinions of the Japanese general public (i.e., Japanese nationals in an inclusive sense – people who are members of the Japanese population [please see ‘Selection of respondents’ in the ‘Materials & methods’ section for more information]) on a wide range of ethical issues regarding the creation of IVD-gametes and their use for research and reproduction. This study aimed to elucidate the extent to which the creation and use of IVD-gametes are considered acceptable by the general public and the expectations and concerns they may have. While some parts of the survey results were showed elsewhere [25], the content reported in this paper comprises the following six parts (see the Supplementary Box 1 for 1–3):

- Fact-based explanation of ‘iPSCs’ and questions regarding the respondents’ degree of awareness and interest
- Fact-based explanation of ‘research for creating sperm/ova from hiPSCs’ and a question regarding the respondents’ degree of awareness
- Explanation of ‘the creation and use of hiPSC-derived sperm/ova’, Figure 1, and a question regarding the respondents’ degree of understanding
- Questions about the respondents’ expectations and concerns regarding ‘the creation and use of hiPSC-derived sperm/ova’
- Questions regarding the respondents’ degree of acceptance of ‘the creation and use of hiPSC-derived sperm/ova’
- Questions regarding respondents’ demographic characteristics.

The distinctive feature of this survey was that the respondents received detailed explanations with a figure on IVG research (Figure 1), and were then asked about their expectations, concerns and degree of acceptance regarding ‘the creation and use of hiPSC-derived sperm/ova’. This was because, to meet the purpose of this survey, which was to grasp the interests of the general public, we considered it important to have the respondents express their attitudes based on accurate information regarding IVD-gametes, a topic with which the respondents were unfamiliar.

The items for the expectations and concerns associated with the creation and use of IVD-gametes were set based on a review of the scientific aspects of IVG technology compiled by the Expert Panel on Bioethics in the Cabinet Office [16] as well as literature in bioethics research. We listed the following 12 items as expectations: elucidate human development and genetic mechanisms (hereafter, elucidation of the mechanism of embryonic development), elucidate causes of infertility attributable to sperm and eggs (hereafter, elucidation of the etiology of infertility), develop therapies or drugs for infertility attributable to sperm or eggs (hereafter, development of therapeutic modalities and drugs for infertility), elucidate the cause of congenital diseases (hereafter, elucidation of the etiology of congenital diseases), develop therapies or drugs for congenital diseases (hereafter, development of therapeutic modalities and drugs for congenital diseases), increase the ease of obtaining sperm and eggs for research (hereafter, availability of gametes for research), increase the ease of obtaining fertilized eggs (embryos) for research (hereafter, availability of embryos for research), enable anyone who desires a child to have a genetically related child (hereafter, birth of genetically related children), increase the ease of obtaining sperm and eggs to be used for reproductive medicine (hereafter, availability of gametes for reproduction), increase the ease of obtaining fertilized eggs (embryos) to be used for reproductive medicine (hereafter, availability of embryos for reproduction), enable curtailment of genetic diseases in society through intensive use of sperm and ova with specific characteristics (hereafter, reduction in genetic diseases), and enable parents to choose the characteristics they desire in their babies (such babies are referred to as designer babies in this paper). For the item of ‘reduction in genetic diseases’, we added the following explanatory note: “Huntington’s disease and some types of hemophilia are caused by genetic abnormalities, and when the parents possess the gene responsible, it is inherited by their children at a certain probability. This item assumes such genetic diseases caused by the abnormalities of specific genes.” We asked the respondents to provide an answer for each item using a 4-point Likert scale (1. Not expected at all; 2. Not expected; 3. Expected; 4. Strongly expected).

Next, we listed the following 16 items as concerns: it is an unnatural process (hereafter, unnaturalness), it impinges on God’s territory (hereafter, playing God), it infringes on human dignity (hereafter, infringement of human dignity), the traditional values of family and reproduction will be lost (hereafter, loss of values), it will generate confusion owing to an undeveloped social system, including family registration and inheritance (hereafter, social turmoil), it will spur the commercialization of reproductive medicine by commodifying sperm and eggs (hereafter, commercialization of reproductive medicine), genetic diversity will be lost owing to intensive use of sperm and eggs with specific characteristics only (hereafter, loss of genetic diversity), the precise physical risks and disadvantages associated with children born through this process are unknown (hereafter, unknown risk to children), the children born through this process will be concerned about their origin and identity (hereafter, infringement of identity), the risks associated with use of these gametes and their effects on the next generation and environment are unknown (hereafter, unknown risk to future generations), it will encourage the tendency to use sperm and eggs as tools (simply as parts of the process) (hereafter, use of gametes as tools), it will also fortify the tendency to use fertilized eggs (embryos) as tools (simply as part of the process) (hereafter, use of embryos as tools), it will promote the destruction of fertilized eggs (embryos) (hereafter, promotion of embryo destruction), it will increase the pressure to have children who are genetically related (hereafter, pressure on parents), it will only be available to limited people, such as wealthy or heterosexual couples (hereafter, limited access to reproduction), and it will lead to the birth of designer babies (children with characteristics desired by the parents [hereafter, designer babies]). Our study presupposes that iPSCs are produced under very strict quality-controlled conditions. We asked the respondents to provide an answer for each item using a 4-point Likert scale (1. Not concerned at all; 2. Not concerned; 3. Concerned; 4. Strongly concerned).

In addition, we framed the following questions to grasp the respondents’ degree of acceptance over a series of processes involving research using IVD-gametes to the clinical use of IVD-gametes:

Regarding ‘the creation and use of sperm/ova derived from hiPSCs’, up to which stage do you feel that you can personally accept it? Please choose the answer that is closest to your opinion.

- Up to the first stage: the creation of sperm/ova from hiPSCs
- Up to the second stage: the creation of fertilized eggs (embryos) from the sperm/ova (derived from hiPSCs)
- Up to the third stage: the birth of children derived from fertilized eggs (embryos) created from sperm/ova (derived from hiPSCs)
- I cannot accept it at all

Owing to the structure of the question, the following possibilities of choice were excluded: the option of allowing the first and third stages (but not allowing the second stage); the option of allowing the second and third stages (but not allowing the first stage); the option of allowing only the second stage (but not allowing the first stage); and the option of allowing only the third stage (and not allowing the first and/or second stage[s]).

Regarding the demographic characteristics, we used the registration data obtained from the survey company for age, sex, marital status and personal income. For other characteristics including educational background, presence or absence of children, religious affiliation and experience of infertility treatment, we set separate questions.

While preparing the questionnaire, we consulted with science communicators who hold PhDs and currently work at a stem cell research institute, regarding the content and readability of the questionnaire. In addition, we asked scientists involved in IVG research to offer advice from a scientific viewpoint regarding the explanations and figures for the creation and use of IVD-gametes as well as the content of the questions.

### Data analysis

To grasp the relationships between the degree of acceptance and the demographic characteristics, expectations, and concerns, simple and multiple regression analyses were performed using the degree of acceptance as an objective variable and each item of the demographic characteristics, expectations and concerns as explanatory variables. In the single regression analysis, the coefficient of determination (R^2^) was regarded as the effect size, and when R^2^ exceeded 0.02, 0.13 or 0.26, it indicates a small effect, middle effect, or large effect, respectively [26]. To avoid multicollinearity in the multiple regression, we estimated the variance inflation factor of each explanation [27,28].

In addition, to identify the characteristics of the respondents who answered ‘I cannot accept it at all’ to the question on the degree of acceptance, binomial logistic regression analysis was performed. In the analysis, the degree of acceptance was used as the objective variable, and respondents were grouped into two groups, those who answered ‘I cannot accept it at all’ and those who answered otherwise.

For the statistical analyses reported herein, we used IBM SPSS Regression 24.0 (IBM Corp., NY, USA) and Microsoft Office Professional Plus 2016 Excel (Microsoft Corp., WA, USA). The significance level was set at p < 0.01, and the effect size was calculated.

## Results

### Demographic characteristics of the respondents

We conducted a survey using the described method and obtained complete answers from 3096 respondents. The respondents’ demographic characteristics are shown in Table 1.

**Table 1.** The demographic characteristics of the respondents (n = 3096).

	n	%
Sex		
Male	1533	49.5
Female	1563	50.5
Age (years)		
20–29	419	13.5
30–39	525	17.0
40–49	593	19.2
50–59	507	16.4
60–69	597	19.3
70–79	455	14.7
Marital status		
Yes, I am married	2066	66.7
No, I am not married	1030	33.3
Presence or absence of children		
Yes, I have children	1871	60.4
No, I do not have children	1225	39.6
Experience of infertility treatment		
No	2772	89.5
Yes	261	8.4
I do not want to answer	63	2.0
Educational background		
Elementary school	4	0.1
Junior high school	75	2.4
High school	1041	33.6
Technical college	351	11.3
Two-year college	327	10.6
Four-year college	1159	37.4
Postgraduate studies (master’s degree)	105	3.4
Postgraduate studies (doctorate)	23	0.7
Other	11	0.4
Personal income (yen/year)		
Less than two million	1175	38
Two to four million	698	22.5
Four to six million	410	13.2
Six to eight million	185	6.0
Eight to ten million	66	2.1
Ten to twelve million	33	1.1
Twelve to fifty million	7	0.2
Fifty to twenty million	6	0.2
Over twenty million	7	0.2
I do not know	195	6.3
I do not want to answer	314	10.1
Religious affiliation		
No, I do not have a religious affiliation	2646	85.5
Yes, I have a religious affiliation	313	10.1
I do not want to answer	137	4.4
How much did you know about iPSCs?		
I knew enough about them to be able to provide an explanation to a certain extent	607	19.6
I had only heard a bit about them	2267	73.2
I was not aware of them	222	7.2
Are you interested in iPSCs?		
Yes, I am interested	2220	71.7
No, I am not interested	876	28.3
How much did you know about the ‘research for creating sperm/ova from hiPSCs’?		
I knew enough about it to be able to provide an explanation to a certain extent	212	6.8
I had only heard a bit about it	1322	42.7
I was not aware of it	1562	50.5
How much did you understand the explanation on ‘the creation and use of hiPSC-derived sperm/ova’?		
I fully understood it	128	4.1
I mostly understood it	2101	67.9
I could not understand it very well	743	24.0
I could not understand it at all	124	4.0

hiPSC: Human-induced pluripotent stem cell; iPSC: Induced pluripotent stem cell.

To the question on the degree of awareness regarding IVG research, 49.5% of respondents answered that they had known about it. 72.0% of the respondents answered that they had understood the explanation regarding the creation and use of IVD-gametes.

### Expectations

**Figure 2.**
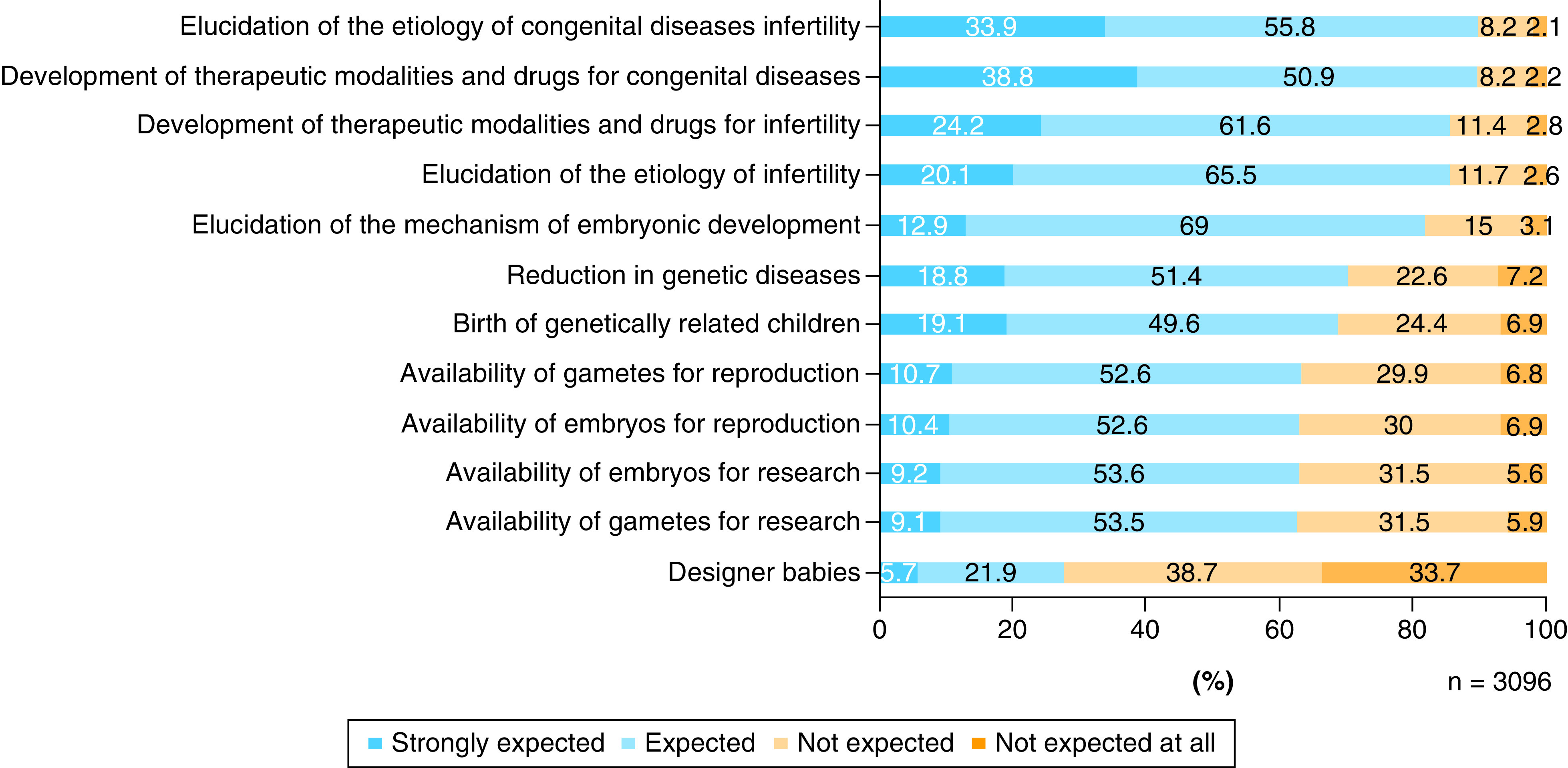
Expectations regarding the creation and use of human-induced pluripotent stem cell-derived sperm/ova.

For each item of expectation, we calculated the sum of the number of respondents who answered ‘Expected’ and the number of those who answered ‘Strongly expected’, and arranged the items in descending order of that sum (Figure 2). The expectations of the respondents were as follows (in descending order): ‘elucidation of etiology of congenital diseases’ (89.7%), ‘development of therapeutic modalities and drugs for congenital diseases’ (89.7%), ‘development of therapeutic modalities and drugs for infertility’ (85.8%), ‘elucidation of etiology of infertility’ (85.6%), ‘elucidation of the mechanism of embryonic development’ (81.9%), ‘reduction in genetic diseases’ (70.2%), ‘birth of genetically related children’ (68.7%), ‘availability of gametes for reproduction’ (63.3%), ‘availability of embryos for reproduction’ (63.0%), ‘availability of embryos for research’ (62.8%), ‘availability of gametes for research’ (62.6%) and ‘designer babies’ (27.6%).

### Concerns

**Figure 3.**
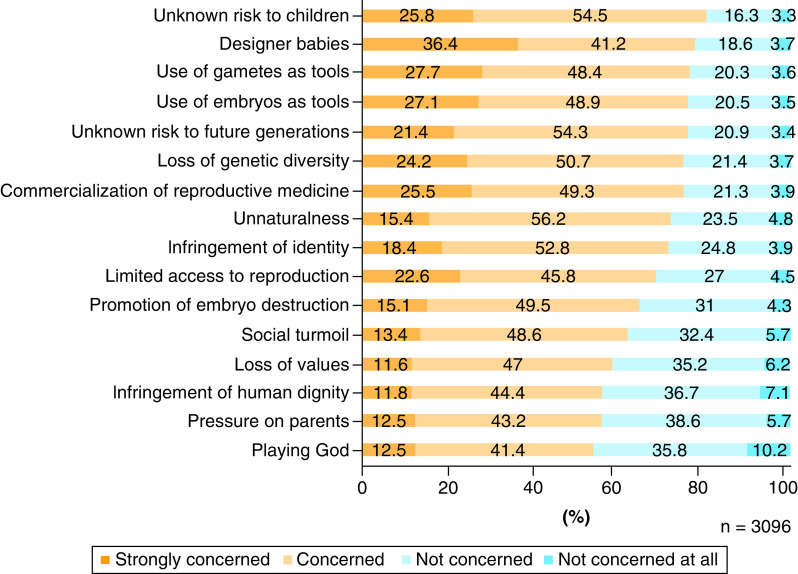
Concerns regarding the creation and use of human-induced pluripotent stem cell-derived sperm/ova.

For each item of concern, we calculated the sum of the number of respondents who answered ‘Concerned’ and the number of those who answered ‘Strongly concerned’, and arranged the items in descending order of that sum (Figure 3). The concerns of the respondents were as follows (in descending order): ‘unknown risk to children’ (80.3%), ‘designer babies’ (77.6%), ‘use of gametes as tools’ (76.1%), ‘use of embryos as tools’ (76.0%), ‘unknown risk to future generations’ (75.7%), ‘loss of genetic diversity’ (74.9%), ‘commercialization of reproductive medicine’ (74.8%), ‘unnaturalness’ (71.6%), ‘infringement of identity’ (71.2%), ‘limited access to reproduction’ (68.4%), ‘promotion of embryo destruction’ (64.6%), ‘social turmoil’ (62.0%), ‘loss of values’ (58.6%), ‘infringement of human dignity’ (56.2%), ‘pressure on parents’ (55.7%) and ‘playing God’ (53.9%). It should be noted that our main objective here was to arrange the items in the order of the degree of concern; therefore, we decided that a nonparametric test was unnecessary.

### Degree of acceptance

**Figure 4.**
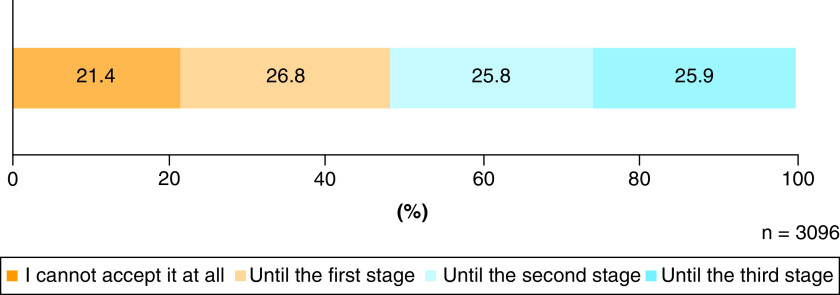
Degree of acceptance regarding the creation and use of human-induced pluripotent stem cell-derived sperm/ova.

Regarding the degree of acceptance of the creation and use of IVD-gametes, 21.4% of the respondents answered that they could not accept it at all, 26.8% answered that they would accept it to the first stage (creation and use of IVD-gametes for research purposes), 25.8% answered that they would accept it to the second stage (creation of and use of embryos with IVD-gametes for research purposes), and 25.9% answered that they would accept it to the third stage (childbirth using embryos with IVD-gametes) (Figure 4). These results indicate that 78.6% of the respondents accepted the creation of IVD-gametes because approving the second or third stage entails approving the first stage. Similarly, these results indicate that 51.7% of the respondents accepted the creation of embryos with IVD-gametes because approving the third stage entails approving the second stage.

Regarding the relationship between the degree of acceptance and demographic characteristics, variables such as age, interest in iPSCs, and degree of understanding of the explanation for the creation and use of IVD-gametes showed statistical significance in multiple regression analysis and a small effect size in a simple regression analysis (p < 0.01, R^2^ >0.02) (Table 2). These results revealed that the respondents with a younger age, a higher degree of interest in iPSCs, and a higher degree of understanding of the explanation for the creation and use of IVD-gametes had a higher degree of acceptance over the series of processes involved in the creation and use of IVD-gametes. It was also revealed that the experience of infertility treatment did not affect attitudes toward the use of IVD-gametes for reproductive purposes (n.s., R^2^ = 0.001).

**Table 2.** Relationship between the iterative acceptance and demographic characteristics of the respondents.

Demographic characteristics	Simple regression analysis^†^						Multiple regression analysis^†^^,^^‡^					
	*β*	95% CI		*t*	p-value	*R^2^*	*β*	95% CI		*t*	p-value	*VIF* ^§^
		*LL*	*UL*					*LL*	*UL*			
Sex	0.056	0.045	0.199	3.112	0.002	0.003	0.066	0.059	0.228	3.335	0.001	1.1
Age	-0.155	-0.013	-0.008	-8.744	0.000	0.024	-0.164	-0.014	-0.008	-7.649	0.000	1.3
Marital status	-0.077	-0.260	-0.097	-4.300	0.000	0.006	-0.041	-0.204	0.014	-1.716	0.086	1.6
Personal income	0.038	0.000	0.066	1.944	0.052	0.001	0.028	-0.008	0.056	1.475	0.140	1.0
Awareness of iPSCs	0.135	0.217	0.369	7.565	0.000	0.018	0.002	-0.093	0.101	0.090	0.929	1.4
Interest in iPSCs	0.190	0.377	0.545	10.780	0.000	0.036	0.139	0.236	0.442	6.457	0.000	1.3
Awareness of AG research	0.079	0.078	0.202	4.424	0.000	0.006	-0.029	-0.127	0.022	-1.382	0.167	1.3
Understanding of the explanation	0.270	0.428	0.551	15.623	0.000	0.073	0.231	0.341	0.500	10.384	0.000	1.4
Educational background	0.040	0.004	0.057	2.250	0.025	0.002	-0.029	-0.051	0.007	-1.464	0.143	1.1
Religious affiliation	0.023	-0.044	0.210	1.278	0.201	0.001	0.029	-0.031	0.243	1.522	0.128	1.0
Children	-0.085	-0.269	-0.112	-4.772	0.000	0.007	-0.019	-0.150	0.063	-0.799	0.425	1.6
Experience of infertility treatment	0.027	-0.032	0.245	1.512	0.131	0.001	0.006	-0.129	0.173	0.292	0.770	1.0

† Respondents who chose ‘I do not want to answer’ for their religious affiliation and their experience of infertility treatment and those who chose ‘I do not know’ or ‘N/A’ for their annual income were excluded from the analyses of the respective demographic characteristics.

‡ The adjusted coefficient of determination, R^2^_adj_ = 0.119.

§ For multiple regression analysis, all explanatory variables were entered using the forced entry method.

β: Standardised partial regression coefficient; iPSC: Induced pluripotent stem cell; LL: Lower limit; R^2^: The coefficient of determination; UL: Upper limit; VIF: Variance inflation factor.

Regarding the relationship between the degree of acceptance and the expectations, variables such as ‘birth of genetically related children’, ‘reduction in genetic diseases’ and ‘designer babies’ showed statistical significance in multiple regression analysis and a small effect size in a simple regression analysis (p < 0.01, R^2^ >0.02) (Table 3). These results revealed that the respondents with those expectations had a higher degree of acceptance over the series of processes involved in the creation and use of IVD-gametes. Similarly, regarding the relationship between the degree of acceptance and the concerns, the variables such as ‘unnaturalness’, ‘playing God’, ‘infringement of human dignity’, ‘loss of values’, ‘unknown risk to children’, ‘promotion of embryo destruction’ and ‘pressure on parents’ showed statistical significance in multiple regression analysis and a small effect size in a simple regression analysis (p < 0.01; R^2^ >0.02) (Table 4). These results revealed that the respondents with those concerns had a lower degree of acceptance of a series over processes involved in the creation and use of IVD-gametes.

**Table 3.** Relationship between the iterative acceptance and the expectations.

Expectations	Simple regression analysis^†^						Multiple regression analysis^†^^,^^‡^					
	*β*	95% CI		*t*	p-value	*R^2^*	*β*	95% CI		*t*	p-value	*VIF* ^§^
		*LL*	*UL*					*LL*	*UL*			
Elucidation of the mechanism of embryonic development	0.237	0.352	0.471	13.544	0.000	0.056	0.009	-0.057	0.087	0.406	0.685	1.7
Elucidation of the etiology of infertility	0.303	0.453	0.566	17.697	0.000	0.092	0.036	-0.040	0.160	1.186	0.236	3.5
Development of therapeutic modalities and drugs for infertility	0.315	0.453	0.560	18.477	0.000	0.099	0.062	0.003	0.195	2.030	0.042	3.5
Elucidation of the etiology of congenital diseases	0.250	0.349	0.459	14.387	0.000	0.063	-0.018	-0.126	0.067	-0.596	0.551	3.5
Development of therapeutic modalities and drugs for congenital diseases	0.269	0.367	0.473	15.510	0.000	0.072	0.074	0.022	0.208	2.416	0.016	3.5
Availability of gametes for research	0.296	0.395	0.496	17.237	0.000	0.088	-0.023	-0.155	0.085	-0.578	0.564	6.3
Availability of embryos for research	0.305	0.411	0.513	17.802	0.000	0.093	0.032	-0.075	0.172	0.765	0.444	6.6
Birth of genetically related children	0.379	0.461	0.547	22.768	0.000	0.144	0.153	0.138	0.268	6.137	0.000	2.3
Availability of gametes for reproduction	0.364	0.479	0.574	21.746	0.000	0.133	0.029	-0.083	0.167	0.657	0.511	7.3
Availability of embryos for reproduction	0.366	0.483	0.578	21.893	0.000	0.134	0.082	-0.005	0.242	1.888	0.059	7.1
Reduction in genetic diseases	0.333	0.401	0.490	19.651	0.000	0.111	0.078	0.045	0.163	3.429	0.001	2.0
Designer babies	0.224	0.233	0.318	12.758	0.000	0.050	0.058	0.027	0.117	3.106	0.002	1.3

† Among the items of expectations, ‘Others’ was excluded from analysis.

‡ The adjusted coefficient of determination, R^2^_adj_ = 0.185.

§ For multiple regression analysis, all explanatory variables were entered using the forced entry method.

β: Standardized partial regression coefficient; LL: Lower limit; R^2^: The coefficient of determination; UL: Upper limit; VIF: Variance inflation factor.

**Table 4.** Relationship between the iterative acceptance and the concerns.

Expectations	Simple regression analysis^†^						Multiple regression analysis^†^^,^^‡^					
	*β*	95% CI		*t*	p-value	*R^2^*	*β*	95% CI		*t*	p-value	*VIF* ^§^
		*LL*	*UL*					*LL*	*UL*			
Unnaturalness	-0.371	-0.594	-0.498	-22.232	0.000	0.138	-0.226	-0.296	-0.156	-6.324	0.000	2.3
Playing God	-0.373	-0.529	-0.443	-22.338	0.000	0.139	-0.115	-0.178	-0.052	-3.597	0.000	2.3
Infringement of human dignity	-0.403	-0.605	-0.515	-24.481	0.000	0.162	-0.230	-0.307	-0.154	-5.880	0.000	3.1
Loss of values	-0.362	-0.563	-0.469	-21.584	0.000	0.131	-0.125	-0.198	-0.051	-3.328	0.001	2.7
Social turmoil	-0.253	-0.408	-0.311	-14.566	0.000	0.064	0.016	-0.045	0.078	0.515	0.607	1.9
Commercialization of reproductive medicine	-0.234	-0.372	-0.277	-13.415	0.000	0.055	-0.004	-0.074	0.066	-0.123	0.902	2.6
Loss of genetic diversity	-0.232	-0.375	-0.278	-13.265	0.000	0.054	0.034	-0.038	0.106	0.928	0.353	2.6
Unknown risk to children	-0.170	-0.301	-0.199	-9.624	0.000	0.029	0.114	0.043	0.185	3.159	0.002	2.4
Infringement of identity	-0.246	-0.405	-0.306	-14.100	0.000	0.060	-0.026	-0.097	0.045	-0.719	0.472	2.4
Unknown risk to future generations	-0.232	-0.390	-0.290	-13.269	0.000	0.054	-0.020	-0.095	0.054	-0.536	0.592	2.6
Use of gametes as tools	-0.231	-0.366	-0.271	-13.188	0.000	0.053	0.013	-0.093	0.119	0.246	0.806	5.9
Use of embryos as tools	-0.234	-0.373	-0.277	-13.373	0.000	0.055	0.050	-0.060	0.159	0.883	0.377	6.3
Promotion of embryo destruction	-0.298	-0.478	-0.381	-17.378	0.000	0.089	-0.130	-0.201	-0.058	-3.555	0.000	2.5
Pressure on parents	-0.281	-0.445	-0.349	-16.284	0.000	0.079	-0.137	-0.199	-0.075	-4.349	0.000	1.9
Limited access to reproduction	-0.150	-0.248	-0.155	-8.430	0.000	0.022	0.057	0.002	0.112	2.014	0.044	1.7
Designer babies	-0.175	-0.276	-0.185	-9.889	0.000	0.031	0.029	-0.027	0.086	1.012	0.312	1.9

† Among the items of concerns, ‘Others’ was excluded from analysis.

‡ The coefficient adjusted of determination, R^2^_adj_ = 0.204.

§ For multiple regression analysis, all explanatory variables were entered using the forced entry method.

β: Standardized partial regression coefficient; LL: Lower limit; R^2^: The coefficient of determination; UL: Upper limit; VIF: Variance inflation factor.

In addition, to identify the demographic characteristics of the respondents who answered ‘I cannot accept it at all’ to the question on the degree of acceptance, binomial logistic regression analysis was performed. Regarding the relationship with the demographic characteristics, ‘interest in iPSCs’ and ‘degree of understanding of the explanation for the creation and use of IVD-gametes’ showed statistical significance in multivariate logistic regression analysis and a small effect size in univariate logistic regression analysis (p < 0.01; R^2^ >0.02) (Table 5). These results indicate that the respondents with a lower degree of interest in iPSCs and a lower degree of understanding of the explanation for the creation and use of IVD-gametes tended to answer that they could not accept the creation and use of IVD-gametes at all. In addition, the expectations such a ‘development of therapeutic modalities and drugs for congenital diseases’ and ‘birth of genetically related children’ showed statistical significance in multivariate analysis and a small effect size in univariate analysis (p < 0.01; R^2^ >0.02) (Table 6). These results indicate that the respondents with expectations such as ‘development of therapeutic modalities and drugs for congenital diseases’ and ‘birth of genetically related children’ tend to accept the creation and use of IVD-gametes at a certain stage. Furthermore, concerns such as ‘unnaturalness’, ‘playing God’, ‘infringement of human dignity’, ‘loss of values’, ‘promotion of embryo destruction’ and ‘pressure on parents’ showed statistical significance in multivariate analysis and a small effect size in univariate analysis (p < 0.01; R^2^ >0.02) (Table 7). These results revealed that the respondents with these concerns tend to answer that they do not accept the creation and use of IVD-gametes at all.

**Table 5.** Demographics characteristics of the respondents who do not accept the creation and use of artificial gametes at all.

Demographic characteristics	Univariate binomial logistic regression analysis^†^									Multivariate binomial logistic regression analysis^†^^,^^‡^							
	*B*	*SE B*	*Wald*	OR	95% CI		*df*	p-value	*R^2^*	*B*	*SE B*	*Wald*	OR	95% CI		*df*	p-value
					*LL*	*UL*								*LL*	*UL*		
Sex	-0.175	0.088	3.976	0.839	0.707	0.997	1	0.046	0.001	-0.197	0.114	2.998	0.821	0.657	1.026	1	0.083
Age	0.018	0.003	41.265	1.018	1.013	1.024	1	0.000	0.013	0.028	0.004	50.291	1.029	1.021	1.037	1	0.000
Marital status	0.166	0.095	3.082	1.181	0.981	1.421	1	0.079	0.001	0.131	0.146	0.809	1.140	0.857	1.517	1	0.368
Personal income	-0.073	0.040	3.376	0.930	0.860	1.005	1	0.066	0.001	-0.068	0.046	2.198	0.934	0.853	1.022	1	0.138
Awareness of iPSCs	-0.887	0.095	87.707	0.412	0.342	0.496	1	0.000	0.029	-0.174	0.135	1.664	0.840	0.645	1.094	1	0.197
Interest in iPSCs	-1.202	0.092	172.567	0.300	0.251	0.360	1	0.000	0.053	-0.966	0.128	56.643	0.381	0.296	0.489	1	0.000
Awareness of AG research	-0.305	0.074	17.006	0.737	0.638	0.852	1	0.000	0.005	0.229	0.107	4.600	1.257	1.020	1.550	1	0.032
Understanding of the explanation	-1.259	0.074	287.029	0.284	0.245	0.329	1	0.000	0.097	-1.080	0.100	116.520	0.340	0.279	0.413	1	0.000
Educational background	-0.150	0.030	24.277	0.861	0.811	0.914	1	0.000	0.008	-0.038	0.040	0.906	0.963	0.891	1.041	1	0.341
Religious affiliation	-0.221	0.156	2.016	0.801	0.590	1.088	1	0.156	0.001	-0.291	0.197	2.194	0.747	0.508	1.099	1	0.139
Children	0.242	0.091	7.067	1.274	1.066	1.524	1	0.008	0.002	0.007	0.144	0.002	1.007	0.760	1.334	1	0.963
Experience of infertility treatment	-0.414	0.178	5.421	0.661	0.466	0.937	1	0.020	0.002	-0.250	0.225	1.240	0.779	0.501	1.210	1	0.265

† Among the items of demographic characteristics, ‘I do not want to answer’ in religious affiliation and experience of infertility treatment was excluded from analysis. In addition, ‘I do not know’ or ‘I do not want to answer’ were also excluded from analysis.

‡ The coefficients of determination, Cox-Snell R^2^ = 0.142, R^2^ = 0.150, Nagelkerke R^2^ = 0.222.

B: Partial regression coefficient; iPSC: Induced pluripotent stem cell; LL: Lower limit; OR: Odds ratio; R^2^: The coefficients of determination; UL: Upper limit.

**Table 6.** Expectations of the respondents who do not accept the creation and use of artificial gametes at all.

Expectations	Univariate binomial logistic regression analysis^†^									Multivariate binomial logistic regression analysis^†^^,^^‡^							
	*B*	*SE B*	*Wald*	OR	95% CI		*df*	p-value	*R^2^*	*B*	*SE B*	*Wald*	OR	95% CI		*df*	p-value
					*LL*	*UL*								*LL*	*UL*		
Elucidation of the mechanism of embryonic development	-0.919	0.071	168.767	0.399	0.347	0.458	1	0.000	0.055	-0.111	0.096	1.324	0.895	0.741	1.081	1	0.250
Elucidation of the etiology of infertility	-1.133	0.073	238.223	0.322	0.279	0.372	1	0.000	0.085	-0.097	0.135	0.522	0.907	0.697	1.181	1	0.470
Development of therapeutic modalities and drugs for infertility	-1.152	0.071	262.765	0.316	0.275	0.363	1	0.000	0.095	-0.315	0.129	5.996	0.730	0.567	0.939	1	0.014
Elucidation of the etiology of congenital diseases	-0.982	0.068	206.622	0.375	0.328	0.428	1	0.000	0.071	-0.065	0.129	0.255	0.937	0.727	1.207	1	0.614
Development of therapeutic modalities and drugs for congenital diseases	-0.999	0.066	230.123	0.368	0.324	0.419	1	0.000	0.080	-0.335	0.123	7.429	0.716	0.562	0.910	1	0.006
Availability of gametes for research	-0.870	0.064	187.510	0.419	0.370	0.475	1	0.000	0.062	0.123	0.163	0.569	1.131	0.822	1.555	1	0.451
Availability of embryos for research	-0.912	0.064	199.882	0.402	0.354	0.456	1	0.000	0.067	-0.204	0.166	1.509	0.815	0.588	1.129	1	0.219
Birth of genetically related children	-0.904	0.057	249.508	0.405	0.362	0.453	1	0.000	0.085	-0.243	0.088	7.649	0.784	0.660	0.932	1	0.006
Availability of gametes for reproduction	-1.017	0.063	259.741	0.362	0.320	0.409	1	0.000	0.090	-0.190	0.165	1.330	0.827	0.599	1.142	1	0.249
Availability of embryos for reproduction	-1.019	0.063	261.056	0.361	0.319	0.408	1	0.000	0.090	-0.277	0.162	2.922	0.758	0.552	1.041	1	0.087
Reduction in genetic diseases	-0.813	0.056	212.001	0.443	0.397	0.495	1	0.000	0.071	-0.154	0.081	3.655	0.857	0.732	1.004	1	0.056
Designer babies	-0.314	0.052	35.796	0.731	0.659	0.810	1	0.000	0.012	0.139	0.069	4.112	1.149	1.005	1.314	1	0.043

† Among the items of expectations, ‘Others’ was excluded from analysis.

‡ The coefficients of determination, Cox-Snell R^2^ = 0.139, R^2^ = 0.143, Nagelkerke R^2^ = 0.214.

B: Partial regression coefficient; LL: Lower limit; OR: Odds ratio; R^2^: The coefficients of determination; UL: Upper limit.

**Table 7.** Concerns of the respondents who do not accept the creation and use of artificial gametes at all.

Concerns	Univariate binomial logistic regression analysis^†^									Multivariate binomial logistic regression analysis^†^^,^^‡^							
	*B*	*SE B*	*Wald*	OR	95% CI		*df*	p-value	*R^2^*	*B*	*SE B*	*Wald*	OR	95% CI		*df*	p-value
					*LL*	*UL*								*LL*	*UL*		
Unnaturalness	0.881	0.069	161.691	2.414	2.107	2.765	1	0.000	0.057	0.472	0.102	21.442	1.604	1.313	1.959	1	0.000
Playing God	0.801	0.059	185.761	2.227	1.985	2.499	1	0.000	0.064	0.228	0.086	6.984	1.256	1.061	1.488	1	0.008
Infringement of human dignity	0.896	0.063	201.337	2.450	2.165	2.773	1	0.000	0.070	0.415	0.109	14.662	1.515	1.225	1.874	1	0.000
Loss of values	0.828	0.064	167.733	2.288	2.018	2.593	1	0.000	0.058	0.341	0.105	10.604	1.407	1.146	1.728	1	0.001
Social turmoil	0.527	0.060	76.375	1.693	1.505	1.906	1	0.000	0.025	-0.025	0.090	0.076	0.975	0.818	1.164	1	0.783
Commercialization of reproductive medicine	0.346	0.058	35.255	1.414	1.261	1.585	1	0.000	0.011	-0.134	0.102	1.707	0.875	0.716	1.069	1	0.191
Loss of genetic diversity	0.377	0.060	40.189	1.458	1.298	1.639	1	0.000	0.013	-0.058	0.107	0.298	0.943	0.765	1.163	1	0.585
Unknown risk to children	0.247	0.061	16.349	1.280	1.136	1.442	1	0.000	0.005	-0.300	0.104	8.256	0.741	0.604	0.909	1	0.004
Infringement of identity	0.436	0.061	50.660	1.547	1.372	1.744	1	0.000	0.016	-0.018	0.105	0.029	0.982	0.799	1.208	1	0.866
Unknown risk to future generations	0.424	0.062	46.427	1.529	1.353	1.727	1	0.000	0.015	0.054	0.112	0.234	1.056	0.848	1.314	1	0.628
Use of gametes as tools	0.314	0.058	29.353	1.369	1.222	1.534	1	0.000	0.009	-0.215	0.146	2.177	0.806	0.606	1.073	1	0.140
Use of embryos as tools	0.359	0.059	37.302	1.432	1.276	1.607	1	0.000	0.012	-0.124	0.155	0.645	0.883	0.652	1.196	1	0.422
Promotion of embryo destruction	0.682	0.063	118.247	1.978	1.749	2.236	1	0.000	0.040	0.439	0.109	16.317	1.551	1.254	1.919	1	0.000
Pressure on parents	0.700	0.061	133.907	2.014	1.789	2.268	1	0.000	0.044	0.430	0.089	23.311	1.537	1.291	1.830	1	0.000
Limited access to reproduction	0.275	0.055	24.523	1.316	1.181	1.467	1	0.000	0.008	-0.056	0.081	0.466	0.946	0.806	1.110	1	0.495
Designer babies	0.130	0.054	5.818	1.139	1.025	1.265	1	0.016	0.002	-0.363	0.083	19.314	0.696	0.592	0.818	1	0.000

† Among the items of concerns, ‘Others’ was excluded from analysis.

‡ The coefficients of determination, Cox-Snell R^2^ = 0.120, R^2^ = 0.123, Nagelkerke R^2^ = 0.185.

B: Partial regression coefficient; LL: Lower limit; OR: Odds ratio; R^2^: The coefficients of determination; UL: Upper limit.

## Discussion

### Characteristics of the respondents who would not accept the creation & use of IVD-gametes at all

In the conventional bioethics debate, there have been no ethical restrictions on the handling of gametes as long as due consideration is given, although some people have raised the issue of the moral status of gametes [29]. In this survey, approximately 80% of the respondents answered that they accepted the creation and use of IVD-gametes for basic research purposes, which is already permitted by the current Japanese research guidelines, whereas as many as approximately 20% of the respondents answered that they could not accept it at all. Based on these results, we examined the characteristics of the respondents who answered that they could not accept the creation and use of IVD-gametes at all. Our results indicated that such respondents fall into either or both of the following categories.

One is the category of people who have a strong antipathy to life manipulation (artificial intervention in reproduction). Those who answered that they could not accept the creation and use of IVD-gametes at all tended not to express the expectations of ‘development of therapeutic modalities and drugs for congenital diseases’ and ‘birth of genetically related children’ (Table 6).

These results suggest that, even if there is a technology that is likely to bring clinical benefits in the future, those people would have no expectation of these benefits and show a strong antipathy toward the development of such technology. They also tended to exhibit the concerns of ‘unnaturalness’, ‘playing God’ and ‘infringement of human dignity’ (Table 7). In a previously conducted survey involving the Japanese general public on the degree of acceptance, expectations, and concerns regarding human–animal chimeric embryo research [30], approximately 20% of the respondents answered that they could not accept the research at all and expressed concerns similar to those observed in the present survey, such as ‘unnaturalness’ and ‘infringement of human dignity’. Those who expressed these concerns regarding a technology and answer that they cannot accept it at all may display the same attitude toward other life-manipulating technologies, which should be verified in future research.

Another is the category of people who have concerns regarding the potential ramifications of IVG technology. Those who answered that they could not accept the creation and use of IVD-gametes at all also tended to express the concerns of ‘loss of values’, ‘promotion of embryo destruction’, ‘unknown risk to children’ and ‘pressure on parents.’ These concerns are associated more with the creation and use of embryos with IVD-gametes for research and reproductive purposes (the second and third stages) than the creation and use of IVD-gametes for research purposes alone (the first stage). Based on these results, their reactions may be explained by the ‘slippery slope argument’, which is often cited in bioethics discussions [31]. The ‘slippery slope argument’ is a way of thinking in which, taking IVG technology as an example, the creation and use of IVD-gametes for research is considered ethically desirable or not morally unjust; however, even the creation and use of IVD-gametes for research should not be permitted because, once this act is allowed, it may result in some morally unjust consequences such as the creation and use of embryos with IVD-gametes for research or reproduction. Therefore, among the respondents who answered that they could not accept the creation and use of IVD-gametes at all, there may be some who do not necessarily oppose the creation and use of IVD-gametes for basic research, but are concerned about its possible development into the creation and use of embryos with IVD-gametes for research and clinical practice in the future.

### Characteristics of the respondents who would accept the creation & use of embryos with IVD-gametes for research purposes

The human embryo is considered to have a special value for various reasons, and conventionally, the moral status of the embryo has been discussed actively in the bioethics debate concerning hESC research [32]. Some people argue that, even in cases where the use of surplus embryos for research purposes is ethically justified, the creation and use of research embryos is not ethically justified because of the concerns regarding infringement of human dignity and conversion of embryos into tools [33]. Furthermore, while some argue that the embryo with IVD-gametes is a mere cell aggregate [10,34], the moral status of the embryo with IVD-gametes is generally considered to be equal to that of the embryo [8,19,35].

The Japanese government has prescribed the human embryo as ‘a sprout of human life’ and has taken the basic position of banning the use of embryos for research [36], but has partially permitted the use of surplus embryos and research embryos for research by making some exceptions. As previously mentioned, it has currently been considered in Japan that the use of research embryos for research is more ethically problematic than the use of surplus embryos for research, and the creation and use of research embryos has been permitted only in research expected to contribute to the advancement in ART and research involving the creation and use of hESCs. Within this context, the results of this survey showing that approximately half of the respondents answered that they would accept the creation of embryos with IVD-gametes, which has not been allowed in the current Japanese research guidelines, is astonishing.

Similar results have been reported in previous studies. In 2005, a research group from Johns Hopkins University conducted a survey involving 2212 American general citizens; although the survey did not address the creation of embryos with IVD-gametes. In that survey, they gave a brief explanation to the respondents that there were arguments both for and against the use of research embryos, not surplus embryos, for hESC research, and then asked the respondents about the degree of acceptance. The opinions of the respondents were divided, with approximately 49% approving and 48% opposing the use of research embryos [37].

In our survey questioning the need for the creation of embryos with IVD-gametes as well, the respondents' opinions were divided. However, attention should be paid to interpreting this result. In our survey, slightly less than half of the respondents opposed the creation of embryos with IVD-gametes, but the number of respondents who expressed concerns regarding the use of gametes as tools, use of embryos as tools, unnaturalness, promoting embryo destruction, infringing human dignity and playing God (Figure 3) exceeded the number of those who opposed the creation of embryos with IVD-gametes (Figure 4). These results suggest that the respondents who accepted the creation and use of embryos with IVD-gametes included not only those who approve it unconditionally but also a certain number of people who take cautious attitudes.

### Characteristics of the respondents who would accept the birth of children derived from embryos with IVD-gametes

As shown by the results on the degree of acceptance, those who accepted the birth of children derived from embryos with IVD-gametes remained at approximately 20% of the respondents (Figure 4). Furthermore, more than 60% of the respondents had expectations related to the reproductive use of IVD-gametes (reduction in genetic diseases, birth of genetically related children, availability of gametes for reproduction and availability of embryos for reproduction) (Figure 2). Although these results seem inconsistent, findings from a separate survey that we asked the same respondents another question indicated that more than 60% approved the use of IVD-gametes for reproduction by couples suffering from infertility who are unable to achieve pregnancy with existing infertility treatments. This implies that the attitudes toward the use of IVD-gametes for reproduction can change depending on the conditions.

Hendriks *et al.* conducted a survey involving 494 couples with infertility who attended a fertility clinic (couples diagnosed with nonobstructive azoospermia and having opted for testicular sperm extraction with intracytoplasmic sperm injection from 2007 to 2012), and obtained the result that approximately 90% of couples opted for the use of IVD-gametes as the first choice or the final means [38]. In our survey, slightly less than 10% of the respondents had experienced infertility treatment; however, the results showed no effect of the experience of infertility treatment on the degree of acceptance (Table 2). As highly involved respondents, we focused on 139 respondents aged 49 years or younger who had a high probability of having undergone fertility treatment in the past several years, and examined their answers regarding the degree of acceptance; 44 (31.7%) answered that they would accept the third stage and 95 (68.3%) answered otherwise. We compared the percentage of those who accepted the third stage among the 139 respondents aged 49 years or younger who had experienced infertility treatment and the percentage of those who accepted the third stage among the other 2957 respondents; but no significant difference was noted in the tendency of answers (χ^2^ = 2.54; df = 1; p = 0.111). In the survey conducted by Hendriks *et al.*, the subjects were limited to couples suffering from infertility who had no chance of conceiving a child if testicular sperm extraction with intracytoplasmic sperm injection failed. This may explain why a higher degree of acceptance of the use of IVD-gametes for reproduction was obtained than with our survey, which did not set such limits and included the respondents who had experienced infertility treatment in general (including the rhythm method and the induction of ovulation) in the analysis.

The structure of the question on the degree of acceptance and concerns regarding the use of IVD-gametes for reproduction were also considered as other factors explaining the relatively small number of respondents who accepted the process until childbirth using IVD-gametes in contrast to the high expectations regarding the use of IVD-gametes for reproduction. In that question, accepting the second stage (the creation and use of embryos with IVD-gametes) was a prerequisite for accepting the third stage (childbirth using embryos with IVD-gametes) over the series of processes involved in the creation and use of IVD-gametes. Therefore, those who would not accept the second stage (creation and use of embryos with IVD-gametes for research purposes) but would accept the third stage had to choose from ‘I cannot accept it at all’ and ‘up to the first stage’. For example, a respondent who accepts childbirth using embryos with IVD-gametes but does not accept the creation and use of embryos with IVD-gametes for research purposes might have exhibited acceptance up to the first stage because this respondent cannot accept the second stage.

In addition, regarding the use of IVD-gametes for reproduction, a large majority of respondents had concerns regarding the unknown risk to future generations and the birth of designer babies. These are also the points that have been highlighted first as ethical problems regarding the use of IVD-gametes for reproduction. These concerns might have been reflected in the small number of respondents who approved the use of embryos with IVD-gametes for reproduction.

### Limitations & significance of our survey

This is the first survey to reveal the degree of acceptance in the general public toward the creation and use of IVD-gametes. Certainly, because the respondents of this survey were monitor members of a survey company, attention should be paid to the fact that the answers do not include the opinions of those who do not usually use the Internet. However, our sampling method was designed to align age and sex with the vital statistics of Japan in order to minimize the bias in answers.

Since this survey was carried out in 2017, the publication of the results only taking place now could be considered a limitation; however, considering that there have been no breakthroughs in IVG technology, nor any policy related debates thereof in Japan, nor any major events that have stirred public interest (such as the sensational news in China about the birth of gene-edited babies [39]), we can safely presume that public awareness of IVG technology has changed very little over the past several years in Japan, and therefore, the time interval between the survey and this study does not significantly affect the results and discussion.

As often noted in bioethics, we should not deduce any normative argument directly from empirical data. In other words, we cannot say that the creation of IVD-gametes should be allowed on the grounds that approximately 80% of respondents approved it, and conversely, we cannot say that the use of IVD-gametes for reproduction should not be allowed from an ethical viewpoint because almost 30% of respondents disapproved it. However, the results of this survey, which partly revealed the interests of the general public, would be useful in promoting the public debate over the ethical/social acceptability of the creation and use of IVD-gametes as well as the ideal way of its regulation in the future.

Furthermore, this survey successfully revealed the attitudes of the general public toward the fertilization of IVD-gametes for research purposes, which is considered to become an issue when the creation of IVD-gametes is achieved in the future. Our results will also be useful in discussing the ideal means of regulating the creation and use of research embryos, including embryos with IVD-gametes.

We understand that IVG research has progressed and that the possibility of IVG technology further developing successfully in the future cannot be denied. The ISSCR 2021 conducted a focus session on ‘The Science and Ethics of Generating Gametes from Stem Cells’ which discussed these issues in detail [40]. Thus, the theme is regarded as important among the community of stem cell researchers, and it seems to be relevant even today. While it is understandable that some skepticism exists as to whether IVG technology will be developed into ART, we believe that it is prudent to consider the ethical and social acceptability of such technology before it is fully developed. Waiting until the technology is complete before considering this would run the risk of not having enough time to discuss its implications before the more practical interests of stakeholders overpower ethical considerations and influence the decision of whether to use it or not. Thus, we believe that the opinions of the general public on IVG will serve as a valuable source of knowledge from which future debates on the subject may greatly benefit; therefore, it is the merit of the current study.

## Conclusion

Considering the possibility of using IVD-gametes for reproduction in the future, international cooperation will be necessary for legal regulation. Conducting similar surveys in other countries in anticipation of such a scenario should reveal the similarities and differences between Japan and other countries in public attitudes toward the creation and use of IVD-gametes. Such international comparison would be the first step toward the establishment of an international regulatory framework that considers the interests of different countries.

**Summary points**

- The creation and use of *in vitro* derived (IVD)-gametes can affect not only individuals but also the whole society and even future generations. Once *in vitro* gametogenesis technology is established, the issue of discussion will be ‘how should IVD-gametes be used ethically in research and clinical settings’ rather than ‘whether the creation and use of IVD-gametes should be permitted’.
- While it is necessary to discuss with potential stakeholders who are likely to be affected by the creation and use of IVD-gametes to what extent they should be permitted before the creation of IVD-gametes is achieved, few attitude surveys have been conducted to investigate this, and no such survey has ever been conducted in Japan.
- In this study, an online survey was conducted in May 2017, with the general public as the target population, and 3096 respondents participated.
- In the questions used to grasp the respondents’ degree of acceptance over a series of processes involving research using IVD-gametes to the clinical use of IVD-gametes, 78.6% of the respondents answered that they would accept the creation and use of IVD-gametes for research purposes, 51.7% answered that they would accept the creation and use of embryos with IVD-gametes for research purposes, and 25.9% answered that they would accept childbirth using embryos with IVD-gametes. In contrast, 21.4% of the respondents answered that they could not accept any of the presented conditions.
- The results of this survey showing that approximately half of the respondents answered that they would accept the creation of embryos with IVD-gametes, which has not been allowed in the current Japanese research guidelines is astonishing.
- While more than 60% of the respondents expressed their expectation for reproductive use of IVD-gametes, only about 20% of the respondents answered that they would allow reproductive use. This could be partly because the structure of the questionnaire and the concerns that respondents had about IVD-gametes may have affected the results of their response.
- Considering the possibility of using IVD-gametes for reproduction in the future, international cooperation will be necessary for legal regulation. Conducting similar surveys in other countries in anticipation of such a scenario should reveal the similarities and differences between Japan and other countries in public attitudes toward the creation and use of IVD-gametes.
