# Supplementary material for: Public attitudes in Japan toward the creation and use of gametes derived from human-induced pluripotent stem cells
Source: Future Sci OA. 2021 Oct 22;7(10):FSO755. doi: 10.2144/fsoa-2021-0066 (PMC8610011; doi:10.2144/fsoa-2021-0066)
Supplement: Supplementary file 1 [file fsoa-07-755-s1.docx]

**Supplementary Box 1**

Explanation regarding induced pluripotent stem cells (iPSCs) and research for creating sperm/ova from human induced pluripotent stem cells (hiPSCs)

1. Induced pluripotent stem cells (iPSCs)

- Human induced pluripotent stem cells (hiPSCs) are the cells created from somatic cells, including those of the skin and the blood, which can be differentiated into any type of cell that constitutes the body.
- Human embryonic stem cells (hESCs), which have the same potential, are generated from fertilized eggs (embryos). At present, fertilized eggs (embryos) that were created for infertility treatment, but were not used and therefore donated to research, have been used for creating hESCs.
- In creating hESCs, the use (destruction) of fertilized eggs (embryos), which are the “emerging potential of human life,” has been considered problematic. The creation and use of hiPSCs has attracted attention as a solution to this problem.
- Regenerative medicine is medicine that involves transplantation of new cells into organs and tissues whose functions are impaired because of accidents or diseases and restores their functions. iPSCs are expected to be applied in regenerative medicine to aid in the development of drugs and to elucidate the etiology of diseases.

Q1. How much did you know about iPSCs?

1. I knew enough about them to be able to provide an explanation to a certain extent.
2. I had only heard about them.
3. I was not aware of them.

Q2. Are you interested in iPSCs?

1. Yes, I am interested.
2. No, I am not interested.
3. Regarding “research for creating sperm/ova from hiPSCs”

- At present, research on the creation of sperm/ova from hiPSCs is in progress.
- To date, it has become possible to create the precursor cells of sperm/ova (called primordial germ cell-like cells) from hiPSCs; however, the technology to create sperm/ova has not yet been established (Supplemental Figure 1).

**
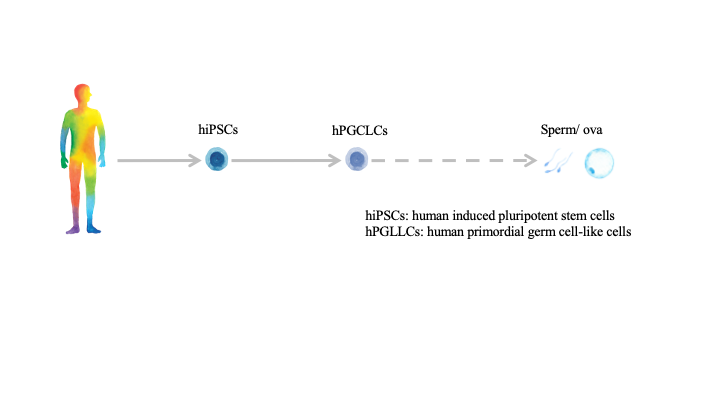
**

< Supplementary Box Figure 1. Process of Creating Sperm/ova from HiPSCs>

Q3. How much did you know about the “research for creating sperm/ova from hiPSCs”?

1. I knew enough about it to be able to provide an explanation to a certain extent.
2. I had only heard about it.
3. I was not aware of it.
4. Regarding “the creation and use of hiPSC-derived sperm/ova”

- The use of hiPSC-derived sperm/ova is expected to help elucidate human evolution, and their application to various studies and treatments is considered promising (Figure II).
- The use of hiPSC-derived sperm/ova is expected to help elucidate the process of sperm/ova formation and to identify the causes of some diseases attributable to sperm/ova.
- The use of hiPSC-derived sperm/ova is expected to aid in the development of therapeutic and testing modalities for infertility and genetic diseases.
- To date, fertilized eggs (embryos) that were created for infertility treatment but were not used (surplus embryos) have been used for research. The use of hiPSC-derived sperm/ova is expected to enable the procurement of fertilized eggs (embryos) without relying on the donation of surplus embryos.
- In the future, hiPSC-derived sperm/ova would be applied to reproductive medicine, enabling couples who could not have children for certain reasons to have genetically related children.
- Even if hiPSC-derived sperm/ova are not used for reproduction, research using them would contribute to the advancement of reproductive medicine.

Furthermore, each stage from the creation of hiPSC-derived sperm/ova through the creation of fertilized eggs (embryos) to childbirth is considered to raise different ethical, legal, and social issues.


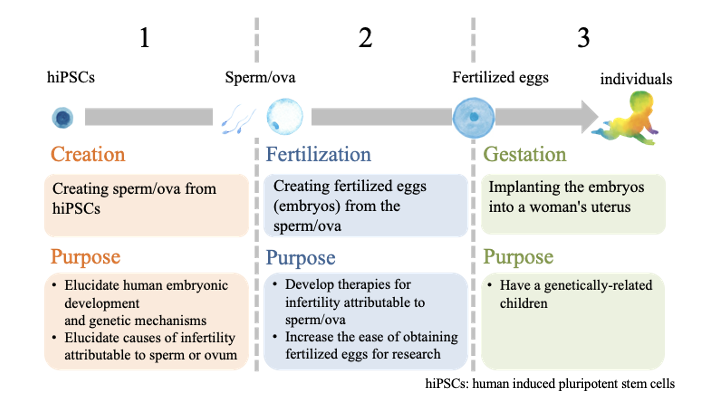


<Supplementary Box Figure 2. Process of Creating and Using HiPSC-derived Sperm/ova>

Q4. How much did you understand the explanation on “the creation and use of hiPSC-derived sperm/ova”?

1. I fully understood it.
2. I mostly understood it.
3. I could not understand it well.
4. I could not understand it at all.
